# Live Cell Imaging of Butterfly Pupal and Larval Wings *In Vivo*

**DOI:** 10.1371/journal.pone.0128332

**Published:** 2015-06-24

**Authors:** Yoshikazu Ohno, Joji M. Otaki

**Affiliations:** The BCPH Unit of Molecular Physiology, Department of Chemistry, Biology and Marine Science, Faculty of Science, University of the Ryukyus, Nishihara, Okinawa, 903–0213, Japan; Oxford Brookes University, UNITED KINGDOM

## Abstract

Butterfly wing color patterns are determined during the late larval and early pupal stages. Characterization of wing epithelial cells at these stages is thus critical to understand how wing structures, including color patterns, are determined. Previously, we successfully recorded real-time *in vivo* images of developing butterfly wings over time at the tissue level. In this study, we employed similar *in vivo* fluorescent imaging techniques to visualize developing wing epithelial cells in the late larval and early pupal stages 1 hour post-pupation. Both larval and pupal epithelial cells were rich in mitochondria and intracellular networks of endoplasmic reticulum, suggesting high metabolic activities, likely in preparation for cellular division, polyploidization, and differentiation. Larval epithelial cells in the wing imaginal disk were relatively large horizontally and tightly packed, whereas pupal epithelial cells were smaller and relatively loosely packed. Furthermore, larval cells were flat, whereas pupal cells were vertically elongated as deep as 130 μm. In pupal cells, many endosome-like or autophagosome-like structures were present in the cellular periphery down to approximately 10 μm in depth, and extensive epidermal feet or filopodia-like processes were observed a few micrometers deep from the cellular surface. Cells were clustered or bundled from approximately 50 μm in depth to deeper levels. From 60 μm to 80 μm in depth, horizontal connections between these clusters were observed. The prospective eyespot and marginal focus areas were resistant to fluorescent dyes, likely because of their non-flat cone-like structures with a relatively thick cuticle. These *in vivo* images provide important information with which to understand processes of epithelial cell differentiation and color pattern determination in butterfly wings.

## Introduction

Diverse and complex butterfly wing color patterns are constructed by regular arrays of microscopic scales that cover the surface of butterfly wings. Scales are extracellular structures produced by epithelial scale cells during the pupal stage [1–3]. Pupal wing tissues are a sac-like structure that consists of the dorsal and ventral epithelial cell sheets, and in between, there is a hemolymph space where hemocytes can move vigorously at the early pupal stage [4]. Scale cells are large polyploidy cells surrounded by smaller epithelial cells, forming a rosette-like structure [5–7]. At the pupal stage, epithelial cells undergo a cell division, forming one daughter precursor that gives rise to scale and socket cells and another daughter cell that undergoes programmed cell death [8–10]. The precursor cells then undergo another cell division, producing scale and socket cells [9,10]. The arrangement of scale arrays also occurs at the pupal stage as an independent process from the determination of color patterns [1,11,12]. Scales of a few different colors are usually grouped as a unit of color pattern on a wing, resulting in macroscopic visual effects. This kind of unit is called a color pattern element [1]. The structure of elements and their relative position on a wing vary, but elements are positioned according to the nymphalid groundplan [1,13–15].

Many butterfly wings harbor an eyespot [16,17], which is a color pattern element that is especially well developed in nymphalid butterflies. Likely because of their concentric structures and their conspicuity, nymphalid eyespots have attracted developmental biologists. Those species that have been used for developmental analyses include the American buckeye, *Junonia coenia* [18–20], the African satyrine butterfly, *Bicyclus anynana* [21–23], the blue pansy, *J*. *orithya* [24–26], and the peacock pansy, *J*. *almana* [27–29]. Many studies have focused on putative molecular mechanisms underlying eyespot development, and they have identified several candidate genes that may be responsible for color pattern formation [30–36]. In these studies, expressed genes were localized at the prospective eyespot using larval imaginal disks. However, functional evidence for these candidate molecules has been scarce. Furthermore, morphological and physiological information on pupal wing tissues has generally been lacking.

Nonetheless, there is solid evidence for the functional activity of eyespot foci from classical surgical studies in which physical damage at the prospective eyespot foci reduces or eliminates eyespots [1,19,21,22,28,37]. That is, the foci of the future eyespots are known to function as organizing centers for color pattern elements. Interestingly enough, these organizing centers are physically marked as pupal cuticle spots at the surface of pupae, probably because of thick cuticle over the organizing cells [37].

We have been studying morphological and physiological aspects of butterfly wing development and color pattern determination [38–40]. To do so at the cellular and tissue levels, we have aimed to build basic descriptive records of normally developing pupal wings. One approach is to analyze the scale number, size, shape, and arrangement in adult wings to infer developmental changes that might have occurred at the pupal stage [41,42]. In addition to these morphometric studies, it is desirable to observe live cells of the larval and pupal wings *in vivo* to accurately record cellular and subcellular morphology. This is especially relevant when developmentally important fine structures may be destroyed by a process of tissue fixation [43,44].

To achieve this goal, we previously developed a simple surgical method to expose a developing hindwing inside a pupal case to allow real-time *in vivo* observations to be made [4,41,45]. Using our surgical technique and state-of-the-art digital imaging technologies, we recorded real-time *in vivo* images of wing tissue development over time in *J*. *orithya* pupae [4]. By mostly focusing on changes at the tissue level, we discovered dynamic movements of the wing system that could not be inferred from fixed specimens [4]. We also reported the abundance of mitochondria in the pupal epithelial cells [4], but the precise characterization of live pupal wings at the cellular level has been lacking.

In the present study, we performed fluorescent observations of the living pupal and larval wing tissues *in vivo* at the cellular and subcellular levels, in contrast to the previous study at the tissue level [4]. We used the latest optical imaging technologies, confocal and multiphoton microscopy systems, to observe differentiating epithelial cells of the pupal and larval wings of *J*. *orithya* and *Zizeeria maha*. We observed structures at the subcellular, cellular, and tissue levels, some of which were not previously known. In contrast to the previous study [4], the present study does not intend to record long-term changes but to record the cellular and subcellular structures of epithelial cells on the wing tissue at the very early pupal stage. However, together with the previous study [4], this descriptive study may pave a way to understanding the physiological aspects of differentiating epithelial cells on butterfly wings.

## Materials and Methods

### Ethics statement

No specific permissions were required to catch and use the blue pansy (peacock pansy) butterfly, *J*. *orithya* (Lepidoptera, Nymphalidae), and the pale grass blue butterfly, *Zizeeria maha* (Lepidoptera, Lycaenidae), for experiments in Okinawa prefecture, Japan, where this study was conducted. These species are two of the most common butterflies in Okinawa prefecture, and they are not endangered or protected species.

### Butterflies

In this study, the blue pansy butterfly, *J*. *orithya*, was mainly used. To obtain eggs, adult females were caught on Okinawa-jima Island (26°00’N−27°00’N, 127°30’E−128°30’E) and Ishigaki-jima Island (24°20’N, 124°9’E). Larvae were occasionally collected from fields. Larvae were reared in a plastic container at ambient temperatures, approximately 27°C, using natural host plants. For experiments, larvae immediately before the prepupal stage that were preparing a base for metamorphosis on the ceiling of a container and pupae immediately after pupation (within 30 min post-pupation after directly watching a pupation process) were subjected to surgical operation, dye loading, and subsequent imaging. Because surgical operation, dye loading, and setting up the imaging system took 10, 30, and 10 min, respectively, the recorded images in this study were from the wing tissues at 1 h post-pupation.

Likewise, adult females of the pale grass blue butterfly, *Z*. *maha*, were caught on Okinawa-jima Island and used only for the experiment to confirm the “deep horizontal connections” in a different species. The pale grass blue butterfly is suitable for observing deep structures, because it is much smaller than the blue pansy butterfly. Thus, fluorescent signals can easily be recovered from relatively deeper levels.

### Surgical operations

**Fig 1:**
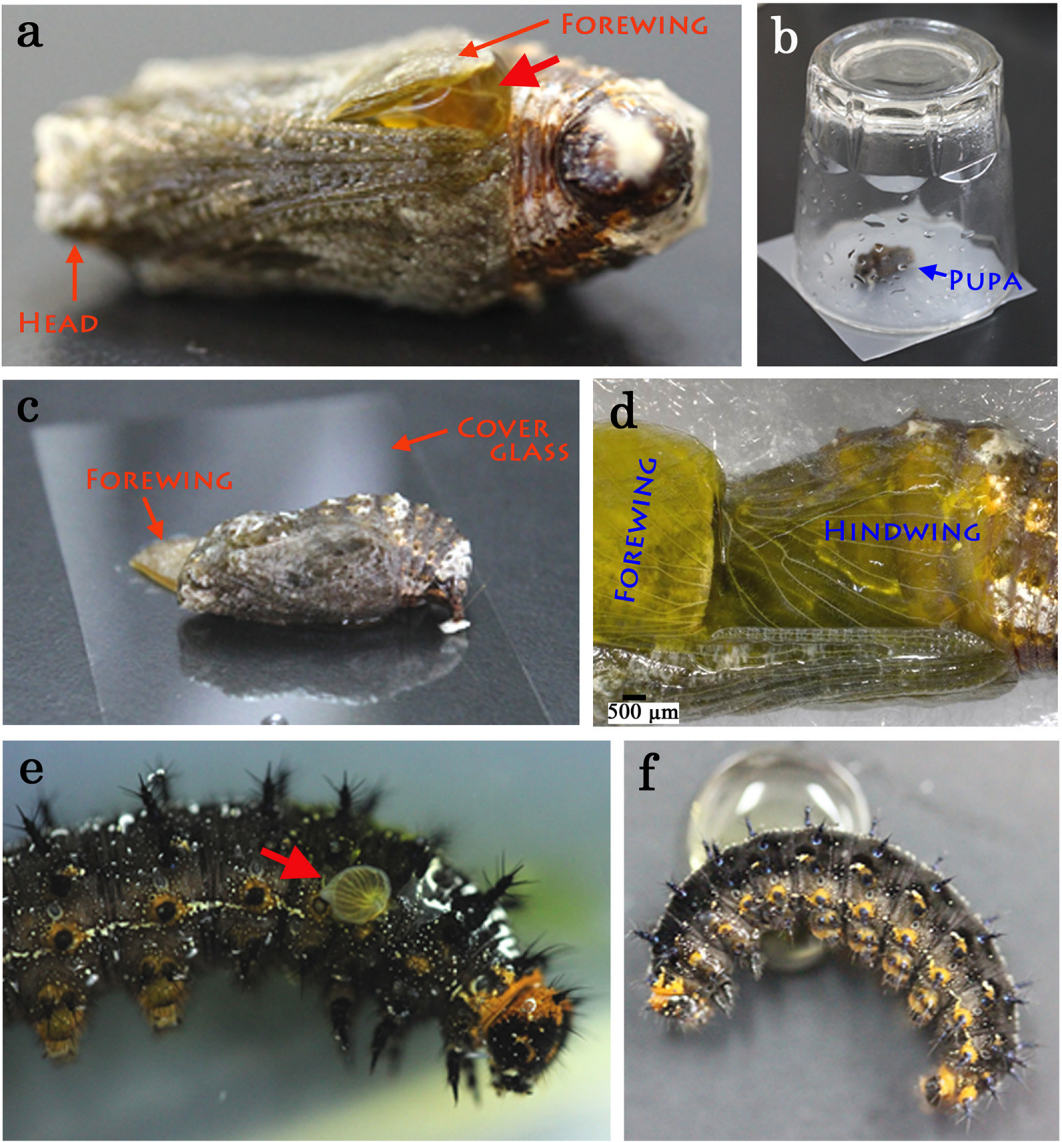
Application of fluorescent dyes to the pupal wing tissue and the larval wing imaginal disk. (**a**) A pupa with a dye-containing solution (a red arrow) sandwiched between wings. The panels a, c, and d are images also shown in a previous study for an introductory purpose. (**b**) A treated pupa in a simple humidified chamber, an upside-down glass cup. (**c**) An operated pupa with its forewing and hindwing on a piece of slide glass. (**d**) Pupal wings seen through a glass slide. (**e**) An operated larva with its wing imaginal disk exposed (a red arrow). (**f**) An operated larva in contact with a dye-containing solution.

The surgical method applied to expose the surfaces of the dorsal hindwing and the ventral forewing of a pupa for *in vivo* observation has been described elsewhere [4,41,45]. Briefly, immediately after pupation (0.5–1 h post-pupation after directly watching a pupation process), the right or left pupal forewing was lifted up using forceps. After a dye loading process (see below), the exposed hindwing and forewing surfaces were placed on a piece of cover glass (0.12–0.17 mm in thickness; Muto Pure Chemicals) to prevent the evaporation of fluid (Fig 1A–1D). Also see Figure A in S1 File. To expose the imaginal disk of a last-instar larva, a small incision was made at the thorax, the disk was exposed, and leaked hemolymph was eliminated immediately, under ice anesthesia (Fig 1E and 1F).

### Fluorescent dyes

We used organelle-specific membrane-permeable fluorescent dyes, Hoechst 33342 (Dojindo Molecular Technologies, Kumamoto, Japan) and SYBR Green I (Life Technologies, Carlsbad, CA, USA) for nuclei, MitoTracker Orange CMTMRos (Life Technologies) for mitochondria, BODIPY FL Thapsigargin (Life Technologies) for endoplasmic reticulum (ER), LysoTracker Red (Life Technologies) for lysosomal bodies, and CFSE (Dojindo) for cytoplasm. CFSE was used for multiphoton microscopy. We also used Rhodamine 123 (Dojindo) for mitochondria, ER Tracker Red (Life Technologies) for ER, and Calcein (Dojindo) for cytoplasm.

A fluorescent dye was dissolved in DMSO (dimethyl sulfoxide) and then added to Insect Ringer’s solution (NaCl 10.93 g, KCl 1.57 g, CaCl_2_·2H_2_O 0.83 g, and MgCl_2_·6H_2_O 0.83 g per liter) to obtain a final concentration. In the case of Hoechst 33342, 1.8 mM aqueous solution (1 mg Hoechst 33342 per 1 mL water) was purchased. Final concentrations of the fluorescent dyes were as follows: 20 μM for Hoechst 33342, 20–100 μM for MitoTracker Orange, 10–20 μM for BODIPY FL Thapsigargin, 1 μM for LysoTracker Red, and 10 μM for CFSE. For SYBR Green I, 5× solution (2,000-fold dilution from the original 10,000× stock solution) was used.

### Dye loading: sandwich method

To load a fluorescent dye into a pupal wing, a loading solution (40 μL or less) was sandwiched between the fore- and hindwings (Fig 1A) in a similar manner to the previously described sandwich method for loading various chemicals [45]. The sandwiched state was maintained in a humidified chamber for 30 min to allow loading at ambient temperature (approximately 27°C) (Fig 1B). After this step, the loading solution was washed off with Insect Ringer’s solution. The forewing was then curled up again, and the hindwing was placed on a piece of cover glass (Fig 1C and 1D). More detailed steps were described in Figure A in S1 File. To load a fluorescent dye into a larval wing imaginal disk, a right wing disk was surgically exposed (Fig 1E) and contacted with the loading solution (Fig 1F). For this surgery, larvae were anesthetized by completely submerging them in water for 0.5–1 h.

### Confocal and multiphoton microscopy systems

We used a real-time confocal imaging system including a Nikon Eclipse Ti-U inverted epifluorescence microscope (Tokyo, Japan), a Yokogawa laser-scanning unit CSU-X1 (Tokyo, Japan), and a Hamamatsu Photonics ImagEM C9100-13 electron-multiplying charge-couple device (EM-CCD) camera (Hamamatsu, Japan). This system was operated by a Hamamatsu Photonics AQUACOSMOS/RATIO system (Hamamatsu, Japan). We obtained *Z*-axis scanning images using a Physik Instrumente P721 PIFCO Piezo Flexure Objective Scanner (Karlsruhe, Germany). We also used another confocal imaging system, a Nikon A1^+^ confocal microscope system (Tokyo, Japan) equipped with high-resolution galvano scanner, operated by the Nikon NIS Elements software (Tokyo, Japan). To obtain images of pupal epithelial scale cells in deep levels, we used a Nikon multiphoton confocal microscope A1 RMP with CFSE. Pupae were anesthetized on ice for the CFSE multiphoton imaging.

When optical image data were acquired by the Nikon A1^+^ confocal system, stacking and cross-section images were constructed by Nikon NIS Elements (Tokyo, Japan). 3D constructions from multiphoton data were also made using the same software with default settings. When optical image data were acquired by the Yokogawa CSU-X1, stacking images were constructed by AQUACOSMOS (Hamamatsu, Japan) and cross-section images by ImageJ (National Institutes of Health, USA).

The excitation and emission filter wavelengths used to obtain these confocal images were as follows: Hoechst 3334 (excitation 405 nm, emission 450/25 nm), SYBR Green I (488 nm, 520/25 nm), MitoTracker Orange (561 nm, 595/25 nm), and BODIPY FL Thapsigargin (488 nm, 525/25 nm). For multiphoton microscopy, the excitation and emission filters for CFSE were 900 nm and 525/25 nm, respectively.

### Size measurements and statistical analysis

Area values of horizontal optical cross sections of epithelial cells at the depth of 1–2 μm were obtained using ImageJ. To do this, 10 well separated cells were randomly chosen from wing epithelial images of a single individual, and this process was repeated for 3 individuals. All area data from 30 cells were used together to calculate mean and standard deviation (SD). Theoretical cell radii (when a cellular cross section forms a circle in an ideal situation) were then calculated according to an equation, *area* = *πr* ^2^. Theoretical cell diameters were then calculated. Cell size difference was examined by two-sided Welch’s *t*-test, using JSTAT 13.0 (Yokohama, Japan). For measuring thickness (apical-basal distance) of larval epithelial cells, stacking images of larval cells stained with MitoTracker Orange were scanned through *z*-axis. Depths of appearance and disappearance of stained mitochondria were recorded, and their subtraction values were considered thickness of epithelial cells. We measured 12 cells from each of 3 individuals. These 36 cells were used to calculate mean and SD.

## Results

### Comparison between larval and pupal wings

**Fig 2:**
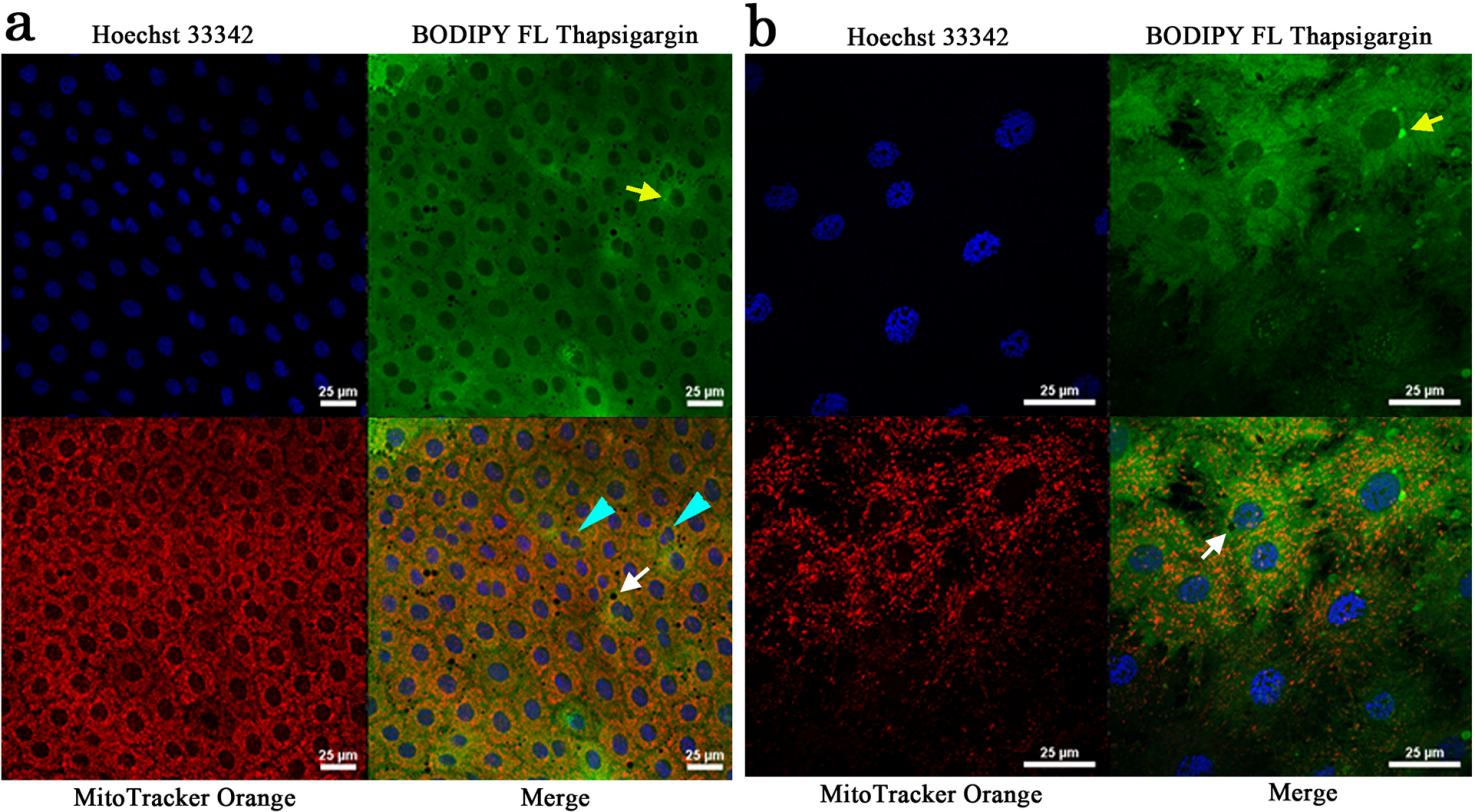
Triple staining of a larval wing imaginal disk. The tissue was stained with Hoechst 33342 for nuclei, BODIPY FL Thapsigargin for ER, and MitoTracker Orange CMTMRos for mitochondria. Yellow arrows indicate peri-nuclear signals. White arrows indicate endosome-like or autophagosome-like unstained structures. Blue arrowheads indicate cells with small double nuclei. (**a**) Low magnification view. (**b**) High magnification view.

**Fig 3:**
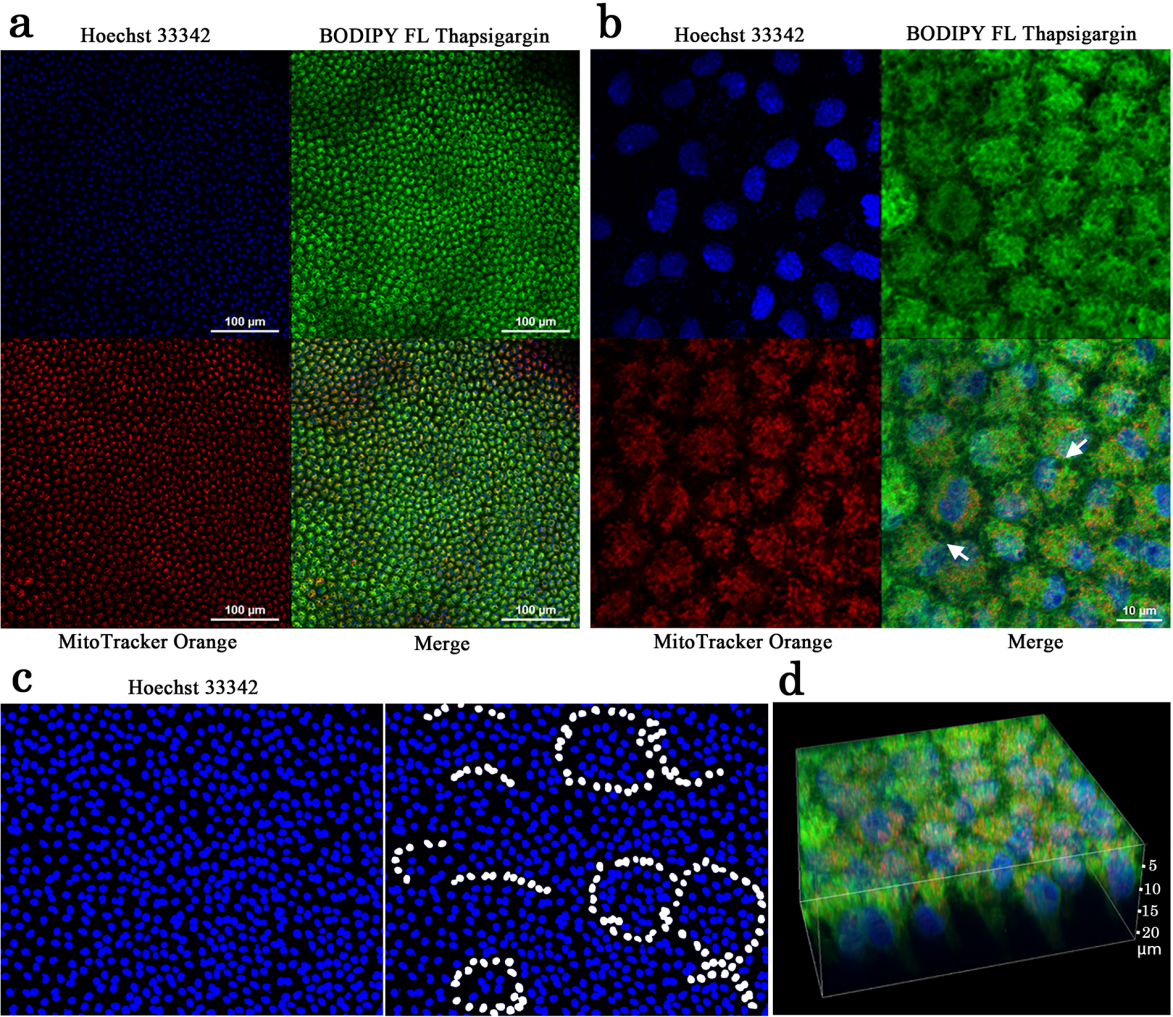
Triple staining of a pupal wing tissue. The tissue was stained with Hoechst 33342 for nuclei, BODIPY FL Thapsigargin for ER, and MitoTracker Orange for mitochondria. White arrows indicate endosome-like or autophagosome-like unstained structures. (**a**) Low magnification view. (**b**) High magnification view. Original scanning images were acquired with 1.0 μm intervals, and those 23 images were stacked to produce the final image of (b) and (d). (**c**) Hoechst 33342 staining. A part of the image shown in (a) was enlarged and its contrast was enhanced. Right and left images are identical except that nuclei with possible circular arrangements are highlighted in white. (**d**) 3D image of an epithelial tissue.

We examined triple staining patterns of the larval wing imaginal disks (*n* = 4; *n* designates the number of individuals observed hereafter) (Fig 2) and the pupal wing tissues (*n* = 4) (Fig 3) using Hoechst 33342 for nuclei, BODIPY FL thapsigargin for ER, and MitoTracker Orange for mitochondria. Although staining patterns were somewhat uneven in the tissue, all three dyes stained the tissues well.

We found both similarities and differences between these larval and pupal tissues. Both larval and pupal cells were rich in mitochondria and ER. Mitochondria and ER staining signals appeared to overlap considerably. This overlap likely stems from the high density of mitochondria and ER networks (see below).

Most larval cells were relatively large along the *xy*-plane, the diameter of which was 31.44 ± 14.88 μm (Fig 2A and 2B). Overall, larval cells were packed relatively tightly. However, some cells were smaller in size or had two small nuclei, likely after a division. ER staining was especially notable in peri-nuclear areas at least in some cells. There were small but distinct endosome-like unstained bodies inside a larval cell.

In contrast, a pupal cell was relatively small at 9.08 ± 4.32 μm in diameter (Fig 3A and 3B). This cell size difference was statistically significant (*t* = 21.97, *df* = 29, *p* < 0.0001; Welch’s *t*-test). Compared with the larval disk, cells were relatively loosely packed. Numerous endosome-like or autophagosome-like unstained bodies were present inside a cell, although their real identity was unclear. They were mostly present in the cellular periphery. Cells had extensive dendritic processes, likely epidermal feet, which made it difficult to identify cellular boundaries between adjacent cells. Interestingly, cells formed rosette-like arrangements (Fig 3C), although it was difficult to identify them clearly at this stage. Furthermore, cells were elongated into deep levels (Fig 3D), although this confocal technique did not allow us to thoroughly determine the deepest structures of these cells.

**Fig 4:**
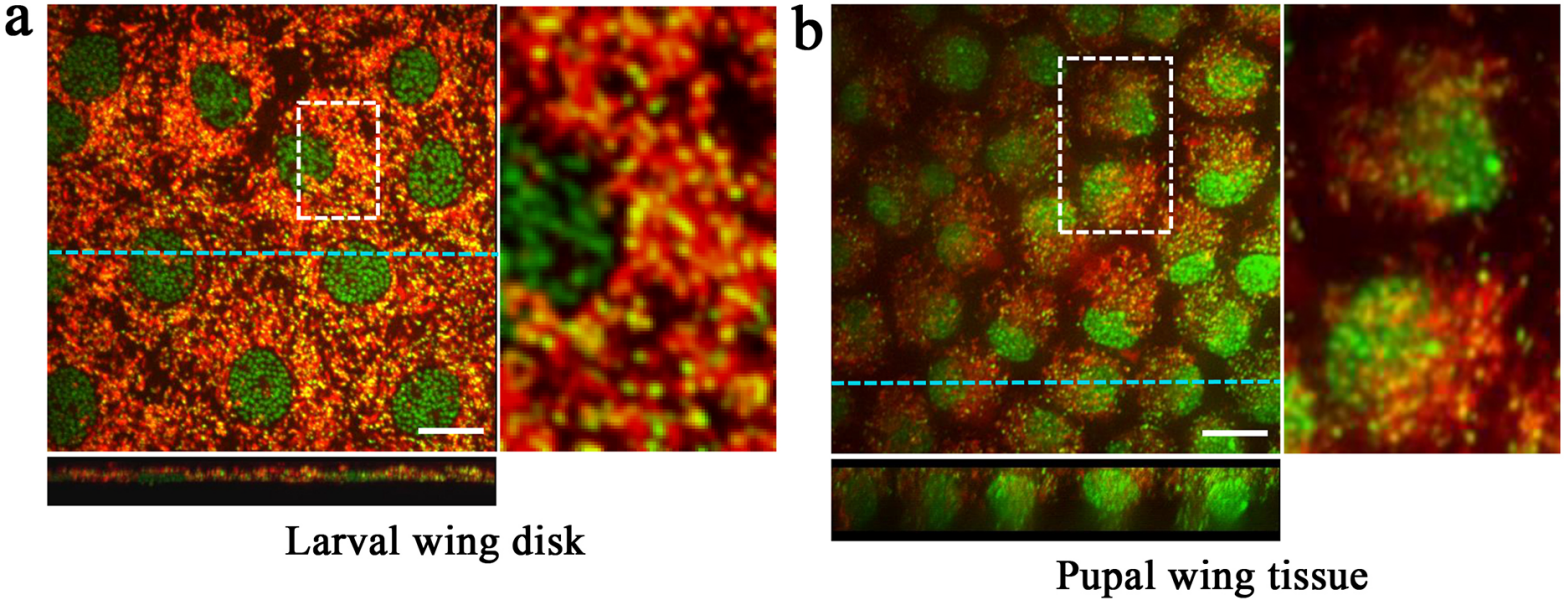
Double staining of a larval wing imaginal disk and pupal wing tissue. The tissue was stained with SYBR Green I for nuclei and MitoTacker Orange for mitochondria. Scale bars indicate 10 μm. (**a**) Larval wing imaginal disk. A portion is enlarged in the right panel. An optical vertical cross-section image shown at the bottom was made along a blue broken line. Thickness of a cell was approximately 2.9 μm. The image was constructed by stacking 28 images down to 5.5 μm in depth with 0.2 μm intervals. (**b**) Pupal wing tissue. The pupal head is positioned to the left. A portion is enlarged in the right panel. An optical vertical cross-section image shown at the bottom was made along a blue broken line. Thickness of a cell was more than 11.4 μm (the deepest portion not detected). The image was constructed by stacking 58 images with 0.2 μm intervals.

In a set of different individuals, we used SYBR Green I and MitoTracker Orange to confirm structural features that were observed above in the larval wing imaginal disk (*n* = 3) (Fig 4A). Larval DNA in nuclei was observed as condensed chromosomes, likely in preparation for cell division. Mitochondrial DNA was clearly identified. Vertical cross-sectioning revealed that larval cells were flat and thin, whose thickness (apical-basal distance) was just 3.25 ± 0.15 μm. As we have already reported staining patterns of the pupal imaginal disk [4], pupal cells also had many mitochondria and were cone-like and thick (*n* = 6) (Fig 4B). Unstained areas between cells were wider in pupal tissues.

### Vertical structures of the pupal epithelial cells

**Fig 5:**
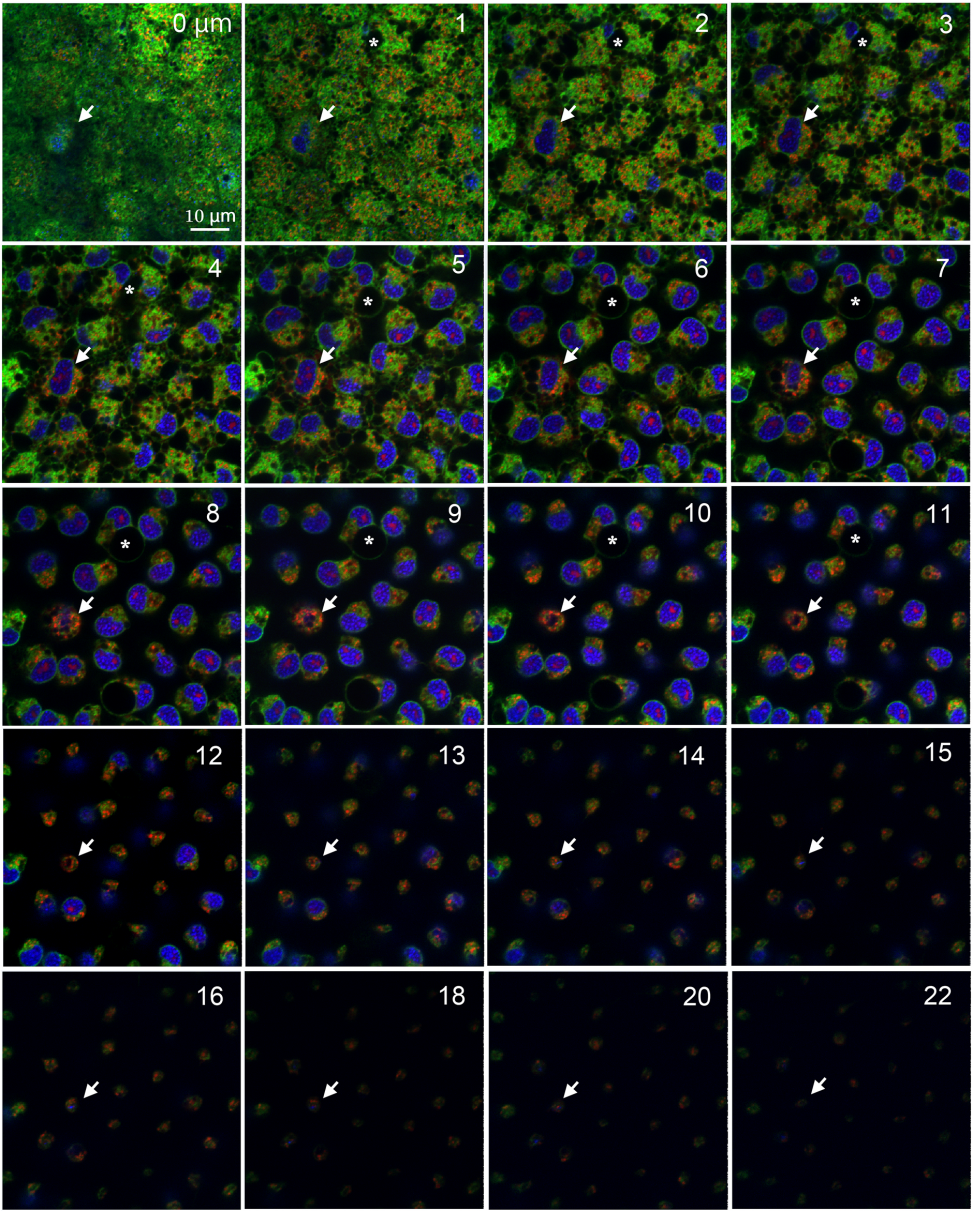
Deep serial cross-sections of pupal epithelial cells triple-stained with Hoechst 33342, BODIPY FL Thapsigargin, and MitoTracker Orange. Numbers at the right upper corner indicate the depth from the apical cellular surface. White arrows indicate a large cell, likely a prospective scale cell. Asterisks indicate a large endosome-like or autophagosome-like unstained structure (see Fig 6). Red dots within a nucleus are not explainable. Also see S1 Movie.

To further characterize the pupal cells, we made detailed optical sections, down to approximately 30 μm from the surface of the tissue using the same set of fluorescent indicators as in Figs 2 and 3 (*n* = 3) (Fig 5; S1 Movie). Epidermal feet were rich down to 5 μm, below which cellular diameters gradually became smaller and the number of epidermal feet connections decreased. The number of endosome-like or autophagosome-like bodies also decreased, but some of them became larger in deep levels at least down to 12 μm. Nuclei were also elongated down to approximately 20 μm in many cells. Indeed, at deep levels, the intracellular space was occupied mostly by a nucleus and endosome-like or autophagosome-like bodies.

Some individual cells had a relatively large nucleus (one of them is shown in Fig 5). This nucleus was located closer to the surface of the tissue than others, and this cell also had more mitochondria than did the others. Based on these observations, they are likely immature scale-forming cells, because scale cells are known to be large polyploidy cells, probably having high metabolic activity [5–7].

**Fig 6:**
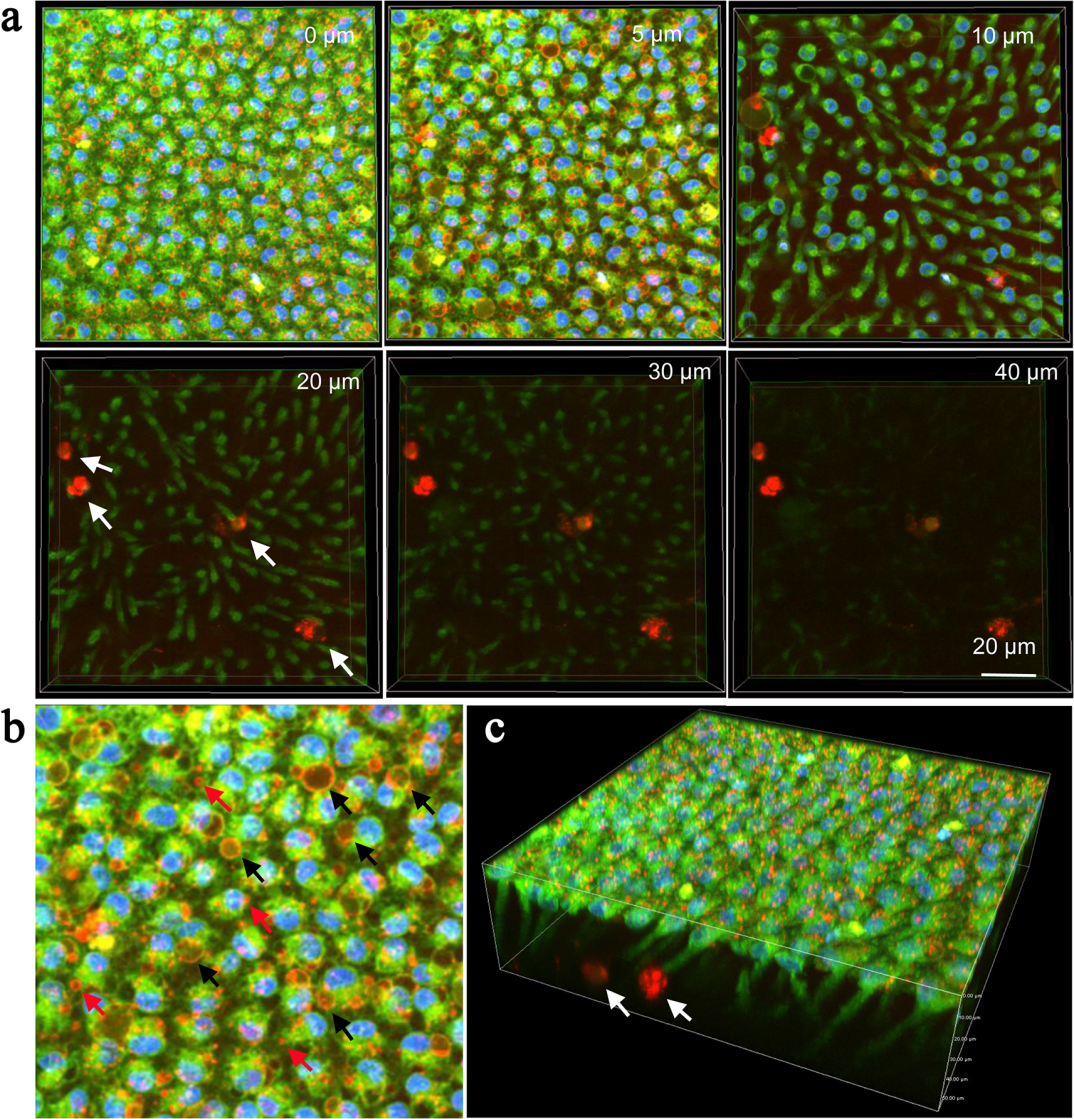
Deep serial cross-sections of pupal epithelial cells triple-stained with Hoechst 33342, BODIPY FL Thapsigargin, and LysoTracker Red. A 3D image was reconstructed first, from which 2D cross-section images were presented in (a) and (b). (**a**) Serial images. Numbers at the right upper corner indicate the depth from the apical cellular surface. Small lysosomal bodies are strongly stained in red. Large endosome-like or autophagosome-like structures are weakly stained in red inside but their membranous structures are seen in orange. Hemocytes are strongly stained in red (arrows). (**b**) Enlarged image of (a), 5 μm in depth. Small lysosomal bodies are indicated by red arrows, whereas endosome-like or autophagosome-like structures are indicated by black arrows. (**c**) 3D image of an epithelial sheet. Stained hemocytes are indicated by arrows.

We also performed fluorescent staining with LysoTracker Red together with BODIPY FL Thapsigargin for ER and Hoechst 33342 for nuclei (*n* = 3) (Fig 6; S2 and S3 Movies). Small lysosomal bodies were numerous at the surface. We confirmed that the endosome-like or autophagosome-like bodies detected previously were also stained with LysoTracker Red mostly on the membrane (Fig 6). Staining levels inside these bodies were generally low. The membranous structures are seen in orange, indicating that they are also stained with BODIPY FL Thapsigargin. Interestingly, LysoTracker Red clearly stained some hemocytes that were located in proximity to epithelial cells. (Fig 6)

**Fig 7:**
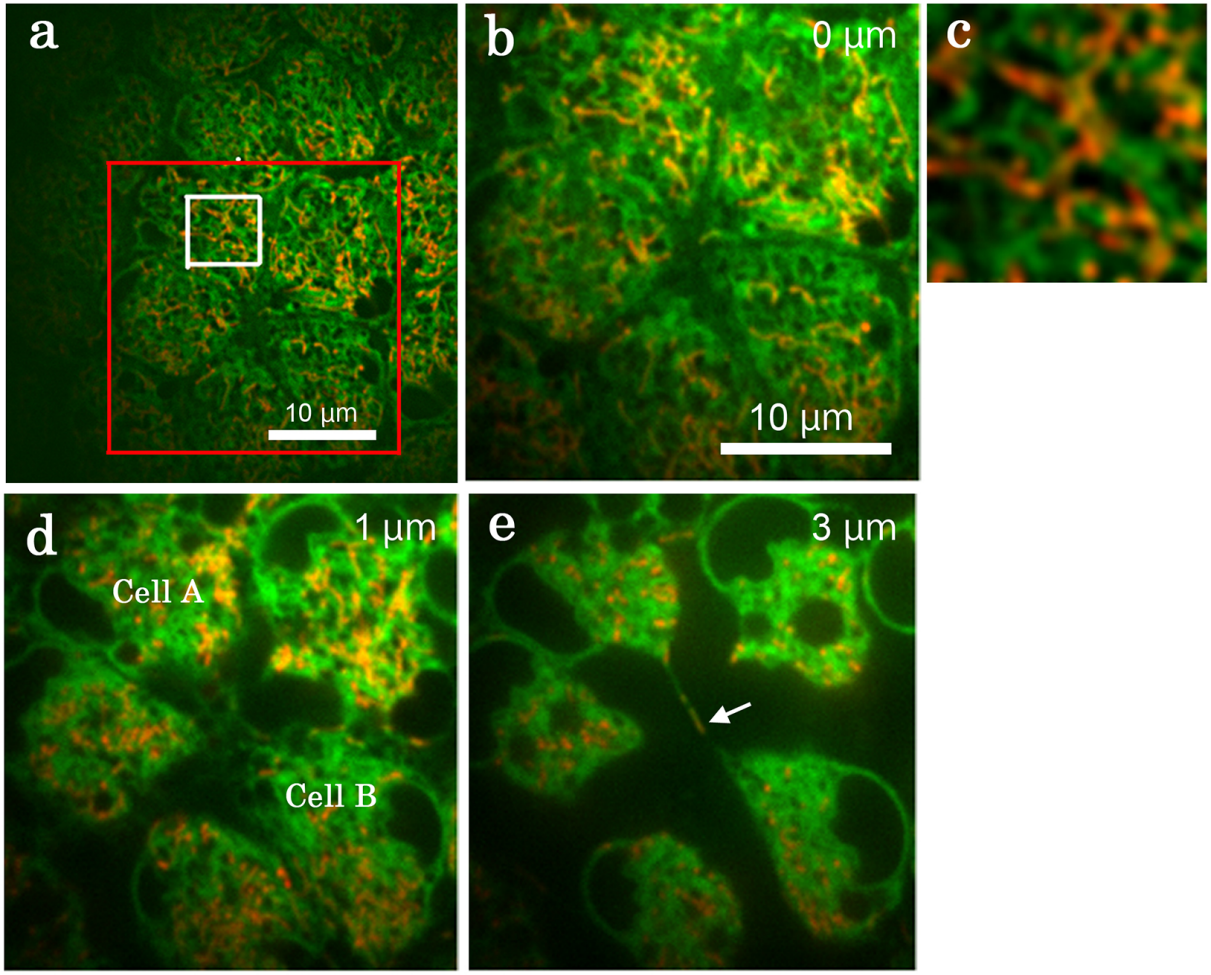
Serial cross-sections of double-stained pupal epithelial cells. Cells were double-stained with BODIPY FL Thapsigargin for ER and MitoTracker Orange for mitochondria. Extensive overlapping of green and red signals is largely because of highly intermingled mitochondria and ER. Cell A and cell B exhibited extensive connections via epidermal feet. A white arrow indicates mitochondria inside an epidermal foot.

In a different set of individuals, we used BODIPY FL Thapsigargin for ER and MitoTracker Orange to confirm structural features that were observed above (*n* = 9) (Fig 7). High spatial resolution images shown here demonstrated that the overlapping mitochondria and ER signals indicate highly intermingled positioning of mitochondria and ER (Fig 7A–7C). We observed extensive intercellular connections between epidermal feet, some of which contained mitochondria (Fig 7D and 7E). It appeared that cells were connected directly by membrane fusion at least in these cases.

We also tested a combination of Rhodamine 123 for mitochondria and ER Tracker Red for ER (*n* = 3), exhibiting overlapping rod-like staining patterns (Figure B in S1 File). Similarly, a combination of Calcein for cytoplasm and MitoTracker Orange for mitochondria also exhibited overlapping rod-like staining patterns (*n* = 5) (Figure B in S1 File).

### Whole 3D structure of epithelial cells

**Fig 8:**
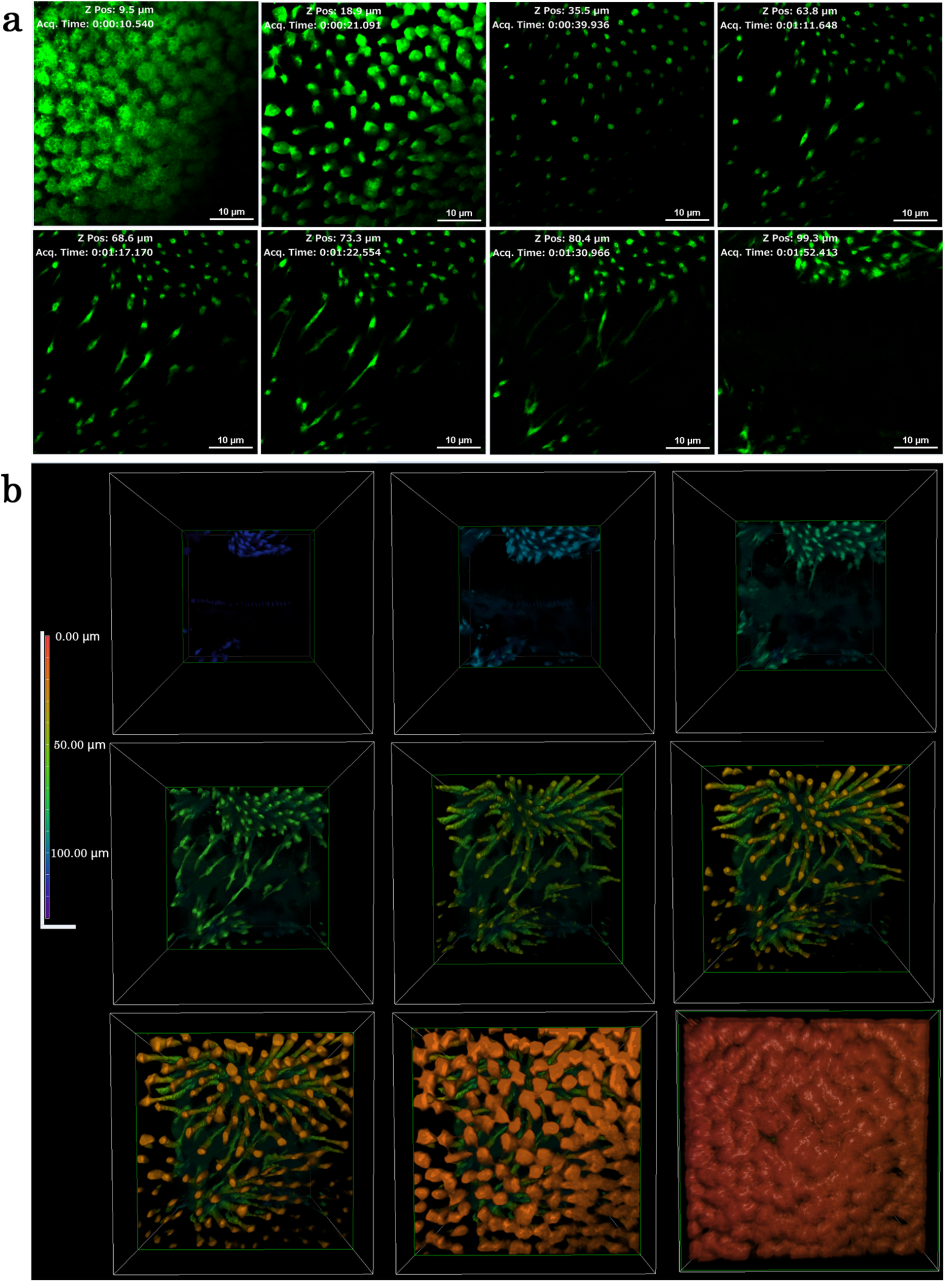
Deep serial cross-sections of pupal epithelial cells and a reconstruction of a stacking 3D image. Cells were stained with CFSE for multiphoton microscopy. (**a**) Sections are presented from the top to the bottom. Also see S2 Movie. (**b**) Reconstruction of a stacking image from the bottom to the top. Also see S3 Movie.

Because the confocal microscopy system we used was not able to cover a depth of more than 30 μm very well, we next used multiphoton microscopy, the focal depth of which is technically much deeper. Using CFSE-stained tissues, we made optical sections deep down to approximately 200 μm, although the deepest structures we observed were approximately 130 μm (*n* = 7) (Fig 8A; S4 Movie). As expected from the results presented in the previous section, epithelial cells were elongated deeply. Down to approximately 40 μm, the cellular diameters became smaller and cells appeared to form clusters or to be bundled, exhibiting dense and less dense areas in the visual field. Interestingly, at approximately 60–80 μm in depth, horizontal connections between clusters appeared. These connections may be cytonemes, which are often difficult to detect in a conventional histochemical analysis [43]. Alternatively, these connections might represent independent cells such as neuronal cells [46] or cells that are not well known in the literature. At approximately 100 μm, the horizontal connections disappeared, but the cellular clusters were distinct.

**Fig 9:**
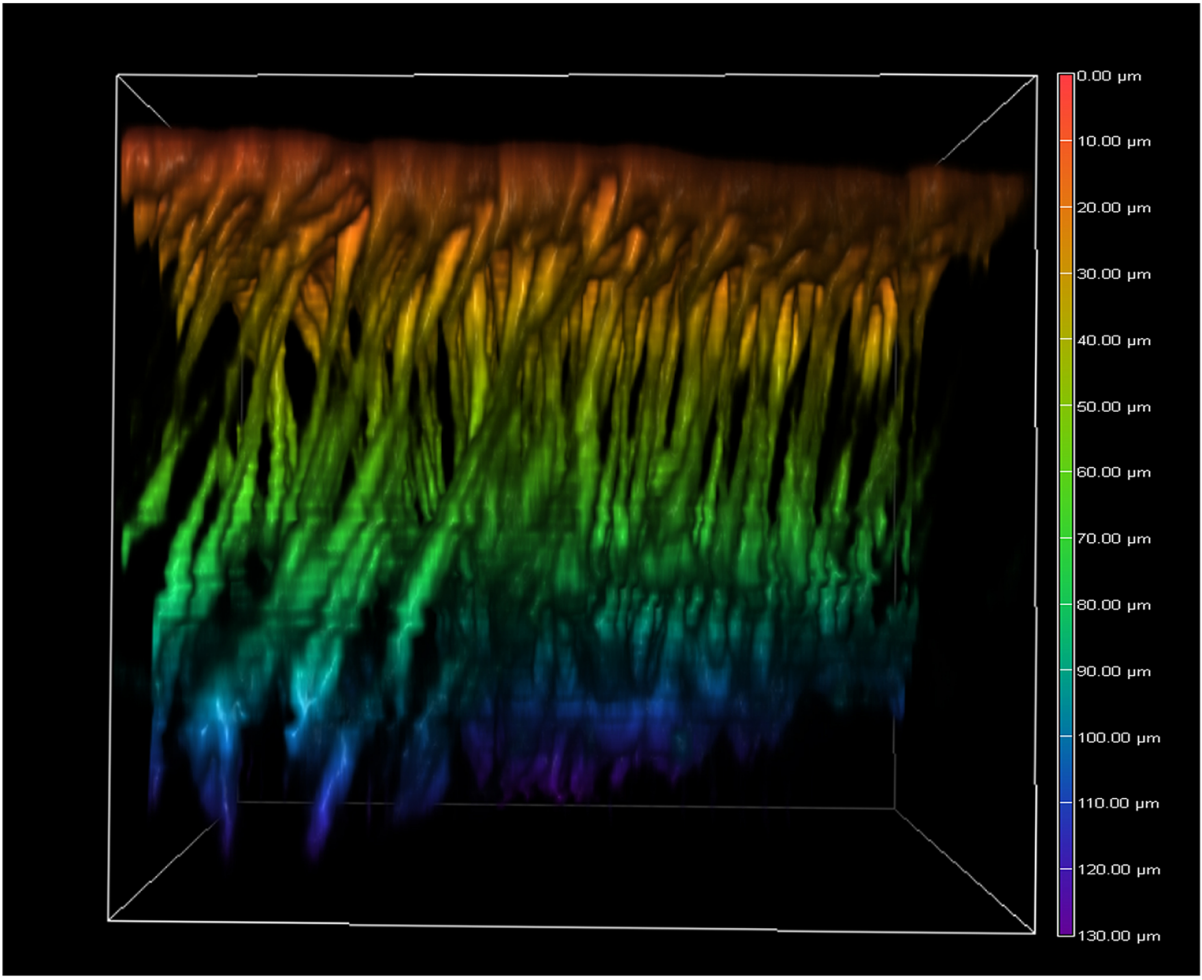
A 3D image of pupal epithelial cells of *J*. *orithya*. An image is shown in a cube with the following dimensions: 143.36 μm (width), 143.36 μm (height), and 130.04 μm (depth). Calibration: 0.28 μm (*x* axis), 0.28 μm (*y* axis), and 2.36 μm (*z* axis). Ripples in deep positions are artifacts caused by sample movement.

We synthesized the optical data above to make stacking images from the bottom to the top (Fig 8B; S5 Movie), and a whole 3D image was reconstructed (Fig 9). Based on these whole structure images, epithelial cells can be described as “cone-like” only down to approximately 20 μm in depth. They may also be described as “elongated” or “neuron-like”.

### Pupal epithelial cells of *Z*. *maha*

**Fig 10:**
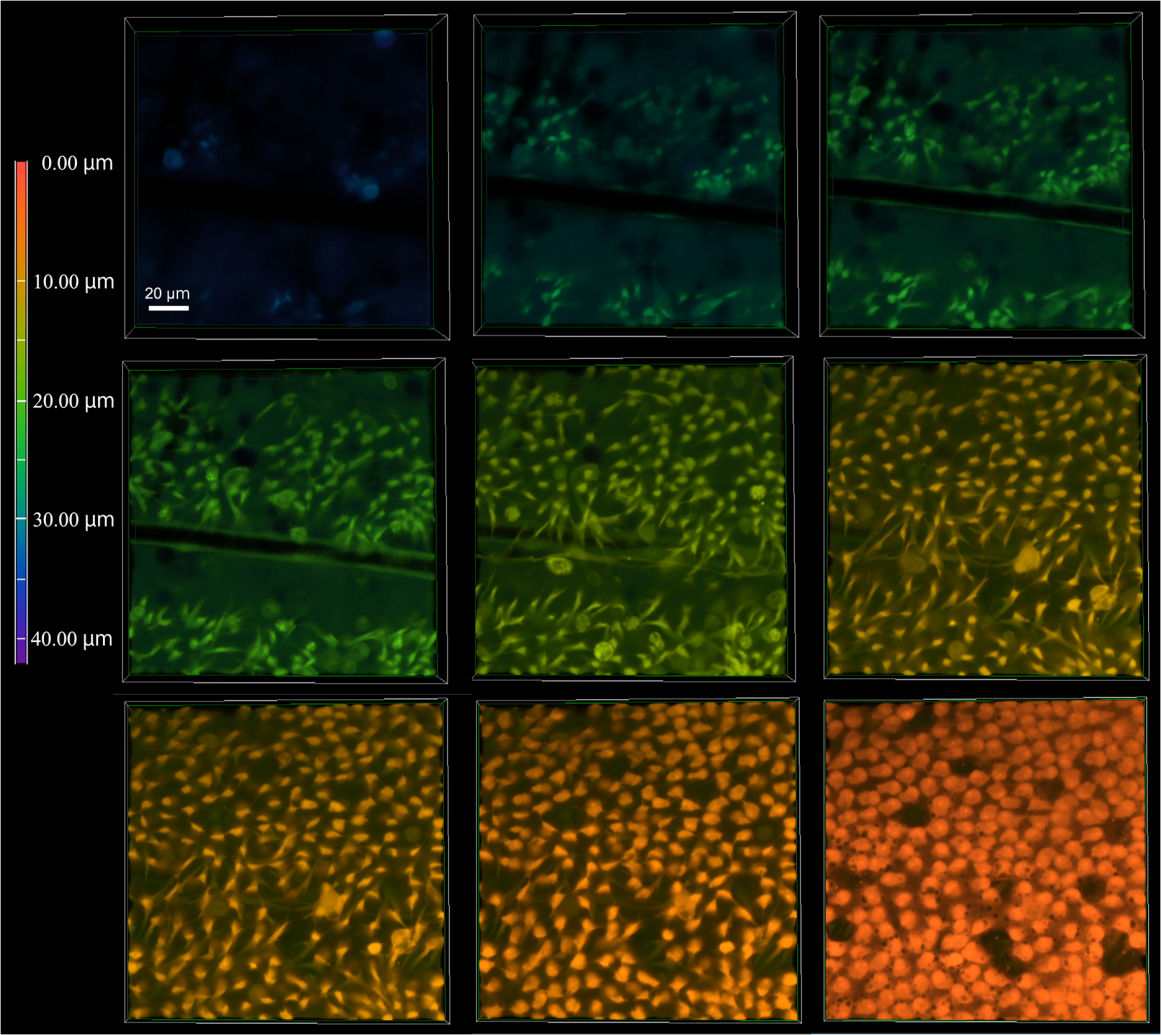
Reconstruction of a stacking 3D image of a pupal wing tissue using a different species, *Z*. *maha*. Reconstruction of a stacking image from the bottom to the top. Cells were stained with CFSE. Also see S6 Movie.

If the deep horizontal connections observed above are cytonemes, they may play a direct role in communications between cells [43]. For example, a dispersion mechanism of morphogenic signaling molecules may be dependent on them. Because of their potential significance in developmental biology, we here presented additional evidence for their existence using a different species, *Z*. *maha*. As expected, CFSE staining revealed that epithelial cells of this species were much smaller; they were as deep as 40 μm. Cellular clusters located in adjacent wing compartments were clearly bridged with the horizontal connections at the deep levels. (*n* = 3) (Fig 10; S6 Movie).

### Macroscopic vertical structures of the pupal wing tissues

**Fig 11:**
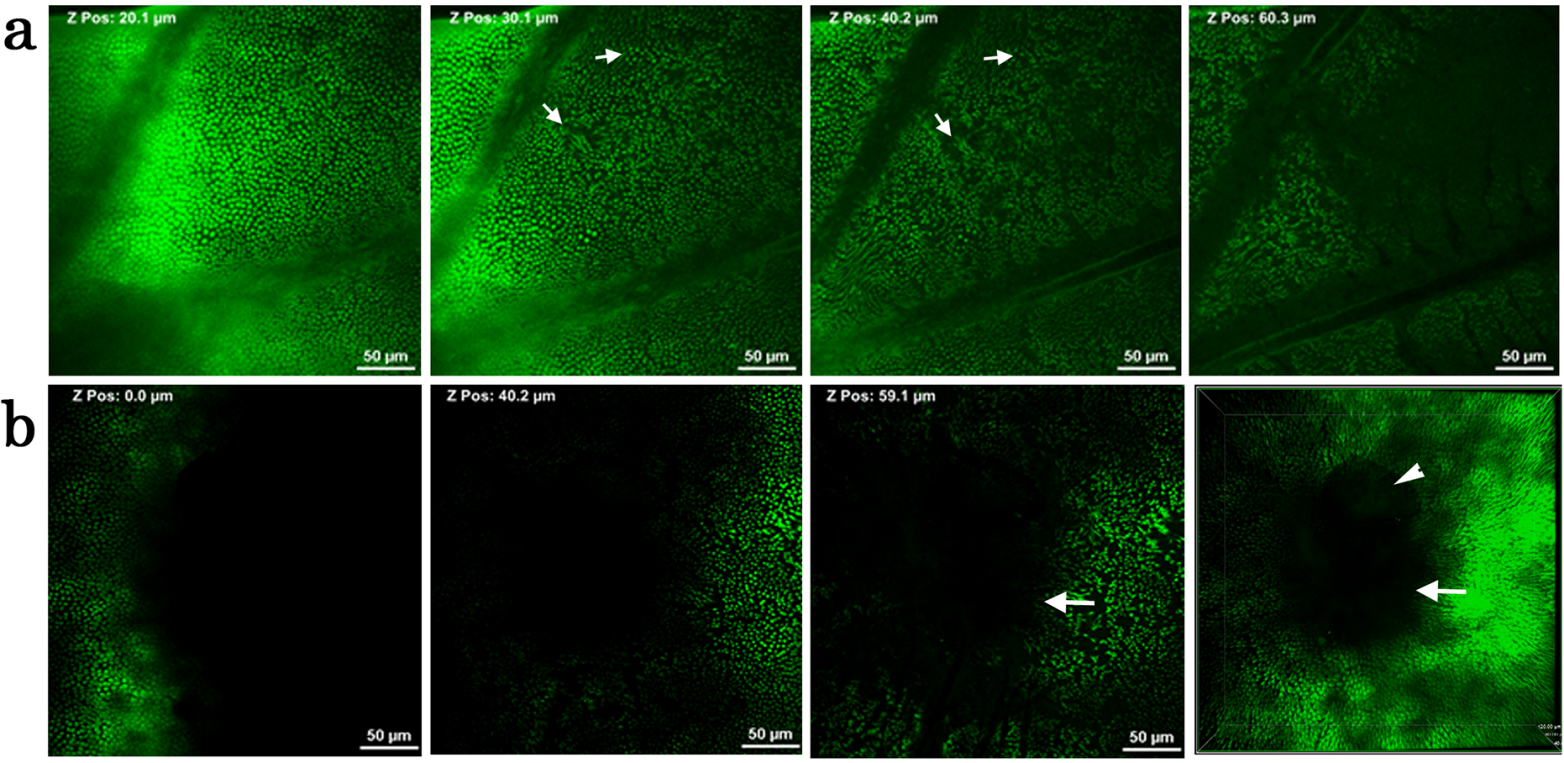
Wide-area images of deep sections of a pupal wing tissue. Cells were stained with CFSE. (**a**) A basal wing area. Possible cellular clusters are indicated by white arrows. (**b**) A prospective eyespot area (white arrows). A bubble is trapped (an arrowhead). The rightmost panel is a synthetic image, showing a cone-like structure.

We also made CFSE-based images that covered a wider area on the wing using multiphoton microscopy (*n* = 3). Elongated epithelial cells appeared to form clusters in a wing basal area (Fig 11A) as well as in a prospective eyespot area (Fig 11B). However, in the prospective eyespot area, images were not clear, likely because of the non-flat cone-like shape of the area that is resistant to staining (Fig 11B).

### Prospective eyespot and marginal focus areas

**Fig 12:**
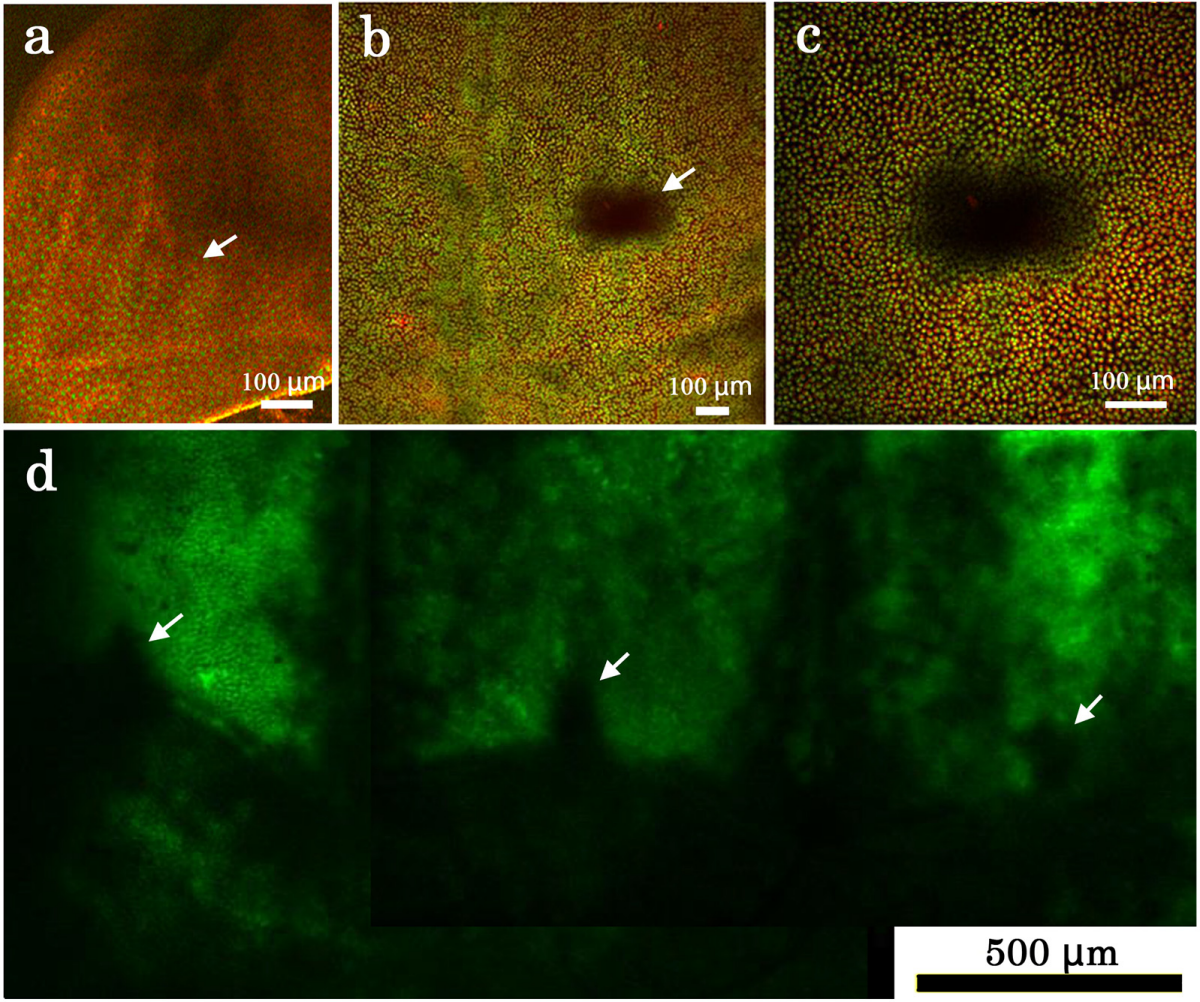
Staining pattern of a prospective eyespot area and edge spots. (**a**) A larval wing imaginal disk. Stained with SYBR Green I for nuclei and MitoTracker Orange for mitochondria. A white arrow indicates a prospective eyespot area, as judged by venation pattern. (**b**) Pupal wing tissue stained with SYBR Green I for nuclei and MitoTracker Orange for mitochondria. A white arrow indicates a prospective eyespot area. (**c**) High magnification of a prospective eyespot area shown in (b). (**d**) Peripheral area of a pupal wing tissue stained with CFSE. Arrowheads indicate pupal edge spots.

We further examined the prospective eyespot area using SYBR Green I and MitoTracker Orange. The larval wing disk did not contain a non-flat unstained structure in the prospective eyespot area (*n* = 3) (Fig 12A), whereas there was such a structure in pupal wing tissue (*n* = 6) (Fig 12B and 12C) as reported in the previous study [4]. The dark appearance here was again likely due to the fact that the fluorescent dyes cannot penetrate well into cells in this area. A different fluorescent dye, CFSE, showed that, in addition to the prospective eyespot area, another organizing center, the marginal focus (or edge spot), was also resistant to staining (Fig 12D).

## Discussion

In this study, we obtained *in vivo* images of larval and pupal cells of butterfly wing tissues, demonstrating the power of fluorescent confocal microscopy in studying living epithelial cells. Most dyes were able to penetrate wing tissue well, although a minor problem was that the staining pattern may be occasionally uneven throughout a wing. This is probably because the surface of the wing tissue is not smooth at the microscopic level. An extreme case is the prospective eyespot, which resists any staining. Heterogeneity in staining was also seen in endosome-like or autophagosome-like bodies. We do not know if this heterogeneity results from technical difficulty for even staining or from biochemical heterogeneity of endosome-like or autophagosome-like bodies. However, precise 3D structures with detailed morphological features especially with deep-rooted elongated structures were revealed for the first time in the present study, because of technical improvement of fluorescent microscopy and butterfly manipulation methods devoid of tissue fixation. Our results are largely consistent with and complementary to those found in classical histological and morphometric studies on lepidopteran wings [1–3,5,6,8–10,46]. On the other hand, we have not recorded dividing pupal wing cells in time-lapse imaging *in vivo*. We have not examined if long-term imaging is possible with these dyes used in this paper except CFSE. CFSE has been known to stay in the cytoplasm for a relatively long time, and indeed, we successfully performed long-term *in vivo* imaging using CFSE, as reported in our previous paper [4]. Time-lapse long-term imaging at the cellular level is a challenge in the future.

Both larval and pupal epithelial cells were rich in mitochondria, ER, and lysosomes at the dorsal surface of the cell. A few combinations of dyes tested here exhibited overlapping staining patterns. We believe that these seemingly overlapping patterns are not dye-dependent, but they arise because of the highly intermingled packaging of mitochondria, ER, and cytoplasm.

Morphological differences between larval and pupal cells are noteworthy. Larval cells were flat, whereas the pupal cells were vertically elongated. The larval cells likely undergo at least one division before becoming pupal wing cells, judging from their size difference. We believe that this dramatic morphological transformation of epithelial cells is likely accompanied by functional changes. We observed that in the early pupal tissue, rosette-like arrangements were emerging, and we identified a prospective scale-forming cell at the center of a rosette-like arrangement. A prospective scale-forming cell had relatively large nucleus that was located at the apical surface, likely because of polyploidization [5–7].

Especially notable at the pupal stage were dense epidermal feet between pupal epithelial cells, which have been proposed to be involved in cell-to-cell communications required for cellular arrangement [1,46–50]. It appears that these cells were physically connected together, not by gap junctions, but directly with fused membranes. Recently, we have recorded long-range slow calcium waves traveling across the pupal wing tissue [51]. The calcium waves could travel via cellular connections including epidermal feet.

Furthermore, in addition to conventional lysosomes, pupal epithelial cells harbored large endosome-like or autophagosome-like bodies. Although their identity is not clear, they may be a special organelle associated with the production of scales. It is interesting to note that some hemocytes were physically close to (probably in contact with) epithelial cells. We speculate that these hemocytes (i.e., macrophage-like cells) are eliminating apoptotic epithelial cells. This is probably why only a small portion of hemocytes that engulfed stained epithelial cells are stained. Note that our sandwich method employed in this experiment primarily stains epithelial cells that have in direct contact with fluorescent dyes. Moreover, because hemocytes are moving cells (and thus direct long-term contact with epithelial cells are unlikely), direct staining of hemocytes with fluorescent dyes that passed through epithelial cells may be unlikely. However, it is also to be noted that some dyes such as LysoTracker Red are highly membrane permeable, which may also be an important physicochemical property to stain hemocytes in any case.

We further performed multiphoton imaging to observe very deep structures of epithelial cells that were not observed in conventional confocal microscopy. To our surprise, we found very long vertical structures of epithelial cells down to 130 μm in the case of *J*. *orithya*. Interestingly, some elongated structures were “clustered” or “bundled” at a certain depth. These cellular clusters may be a unit of differentiation. Another surprising finding was the possible discovery of horizontal bridges that connect two clusters of epithelial tails. These deep horizontal connections differ from epidermal feet, which are found in more shallow positions. We also confirmed these deep horizontal structures in the *Z*. *maha* systems, excluding a possibility of artifacts. To be sure, it is to be noted that, in any butterfly wing system, a possible artifact may arise when the dorsal and ventral epithelial sheets come together tightly. However, this apression process occurs much later [4]. At the time point of 1 h post-pupation, the wing tissue is sac-like, containing much hemolymph between them.

It appears that the deep horizontal connections are likely to be cytonemes [43] or neuronal cells that were observed before [46]. Because fluorescent dyes are unlikely to easily cross apical cells to stain secondary cells located at the deeper levels (with the exception of hemocytes that possibly engulf apoptotic cells), the former possibility is more likely. If so, the deep structures we observed here may be a site that disperses morphogenic signaling molecules that determine wing color patterns [46–50]. We believe that calcium waves travel more shallow positions as discussed above [51]. Thus, differentiating epithelial cells likely use at least two modes of dispersing morphogenic signals of two different qualities throughout a pupal wing tissue.

We confirmed the existence of the deep structures using a smaller butterfly species, *Z*. *maha*. As expected, wing epithelial cells were smaller, and clusters were bridged with horizontal connections. However, we also noticed that there are many species differences between *J*. *orithya* and *Z*. *maha*, including cell shape, surface structure, and organelles. More systematic characterization of the *Z*. *maha* system and its comparison to the *J*. *orithya* system are expected in the future. It is to be noted that *Z*. *maha* has been useful as the lycaenid model system for physiology, genetics, and other fields of biological sciences [52,53].

At the tissue level, the sensitivity of the prospective eyespot focus to all fluorescent dyes was different from other regions. The autofluorescent character of the prospective eyespot areas have been reported to be different from other parts of a wing in the previous study [4]. These results suggest the presence of thick cuticle in the prospective eyespot areas, which is similar to the pupal cuticle spots of the forewing [4]. Another organizing center for the marginal band system, called the marginal focus or edge spot [15,37], also exhibited a similar character as shown in this study and also in the previous study [4]. Therefore, thick cuticles with non-flat structures may be a general feature of organizing centers for color pattern elements. It may be confusing that several papers examined the prospective eyespots by immunohistochemistry or by *in situ* hybridization histochemistry, which require penetration of reagents to the fixed tissues. In these studies, larval wing disks are used. As shown in this paper, there is no physical structures that correspond to the prospective eyespots in the larval wing disks.

In the previous study, we showed that the arrangement of the cellular rows begins at approximately 20 h post-pupation. It is interesting to monitor that time window to examine what occurs at the cellular and subcellular levels. By that time, positional information has already been supplied from organizing centers and the prospective scale cells have committed to their fate regarding scale coloration, size, and shape [41,42]. Scale size (and thus cell size) likely reflects the degree of polyploidy in the epithelial cells of butterflies [1,5–7]. Immediately after pupation, a relatively large cell is present at the center of rosette-like cellular arrangement in *J*. *orithya*, as shown in this paper. We believe, as discussed in the previous paper [4], that cell size and shape may be determined by positional information, which may be identical to a ploidy signal [42]. This line of discussion is reminiscent of the differential adhesiveness model for cellular arrangement and differentiation based on non-diffusible interactions between cells [54].

In the present study, we have established a method to observe live epithelial cells and their detailed structures *in vivo*. We have previously established a gene transfer method for pupal wing cells via a baculovirus vector [55]. Another method to transfer foreign genes, based on electroporation, was also invented [56]. In the future, combinations of these methods will allow us to manipulate molecules inside the epithelium and to monitor changes in living pupal wing cells *in vivo*.

## Supporting Information

S1 File How to perform dye loading to a pupal wing tissue by the sandwich method and Additional double staining patterns of pupal epithelial cells. (PDF)

S1 Movie Optical sections of pupal wing epithelial cells triple-stained with Hoechst 33342 for nuclei, MitoTracker Orange for mitochondria, and BODIPY FL Thapsigargin for ER. (MP4)

S2 Movie Stacking reconstruction image of pupal wing epithelial cells triple-stained with Hoechst 33342 for nuclei, LysoTracker Red for lysosomal bodies, and BODIPY FL Thapsigargin for ER. (MP4)

S3 Movie Vertical sections of a stacking reconstruction image of pupal wing epithelial cells triple-stained with Hoechst 33342 for nuclei, LysoTracker Red for lysosomal bodies, and BODIPY FL Thapsigargin for ER. (MP4)

S4 Movie Optical sections of pupal wing epithelial cells stained with CFSE. (MP4)

S5 Movie Stacking reconstruction image of pupal wing epithelial cells of *J*. *orithya*. (MP4)

S6 Movie Stacking reconstruction image of pupal wing epithelial cells using a different species, *Z*. *maha*. (MP4)
