# Supplementary material for: Live Cell Imaging of Butterfly Pupal and Larval Wings In Vivo
Source: PLoS One. 2015 Jun 24;10(6):e0128332. doi: 10.1371/journal.pone.0128332 (PMC4481267; doi:10.1371/journal.pone.0128332)
Supplement: S1 File — (PDF) [file pone.0128332.s001.pdf]

PLOS ONE

Supporting Information

Live Cell Imaging of Butterfly Pupal and Larval Wings *In Vivo*

Yoshikazu Ohno, Joji M. Otaki

The BCPH Unit of Molecular Physiology,
Department of Chemistry, Biology and Marine Science,
Faculty of Science,
University of the Ryukyus,
Nishihara, Okinawa 903-0213,
Japan

S1 File. How to perform dye loading to a pupal wing tissue by the sandwich method (Figure A) and Additional double staining patterns of pupal epithelial cells (Figure B).

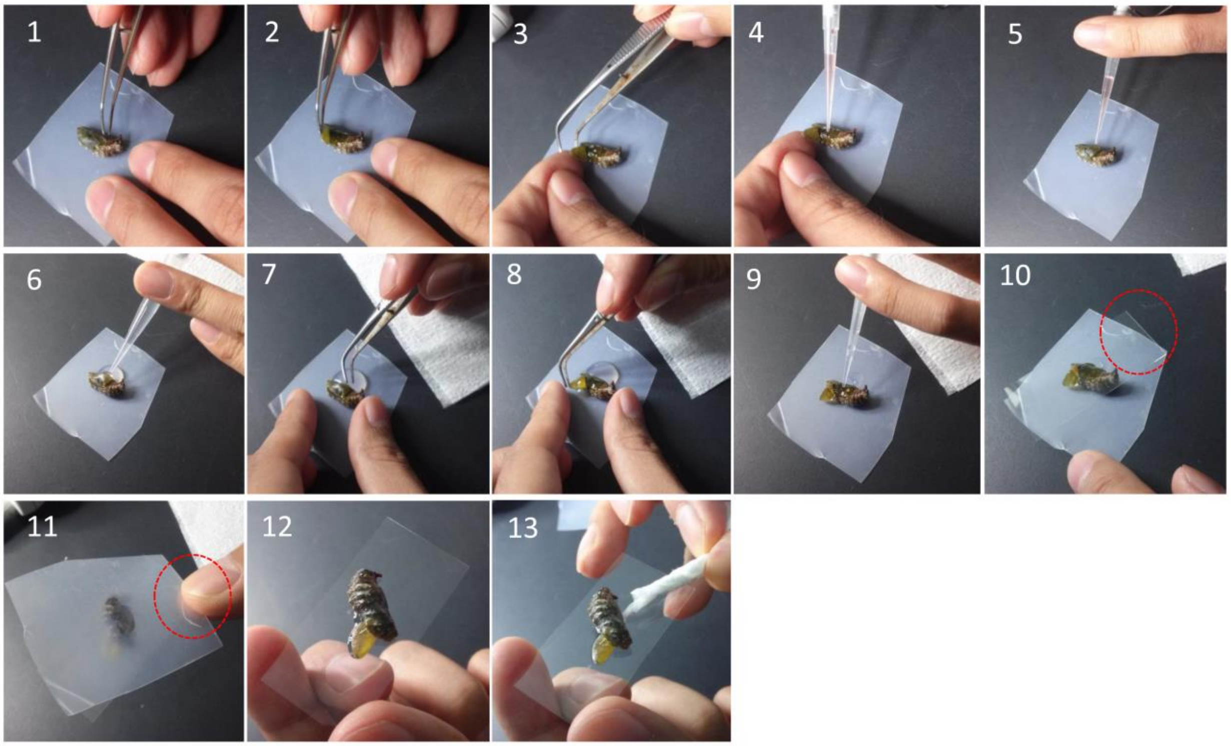

Figure A. How to perform dye loading to a pupal wing tissue by the sandwich method. Various small chemicals including many kinds of fluorescent dyes and inorganic salts may be loaded successfully, although we have not successfully transferred plasmid DNA. This sandwich method, which is based on the wing flipping method invented in 2009 for observing developmental changes *in vivo* [1], has already been utilized in the previous studies [2-4]. Furthermore, this wing flipping method has been applied to an electroporation method for gene transfer in *Bombyx mori* [5]. Readers are also encouraged to refer to these papers. Detailed steps are described below.

(1) A freshly pupated pupa is placed on a piece of plastic film (e.g., Parafilm M®) approximately 30 min after pupation. The tip of the right forewing is carefully picked up with forceps. To do so, recognize wing edges by naked eyes and do not pick up or damage other parts of the body.

(2) The right forewing is slowly picked up in a direction from peripheral to basal portions to expose the hindwing surface. The forewing should be flipped, preferably to the wing base.

(3) The flipped forewing is held vertically with a tip of the index finger. If the forewing is more angled, the wing base will bleed. Such an individual is not usable anymore.

(4) Approximately 20 μL of chemical solution (e.g., fluorescent dye solution for confocal imaging studies) is applied onto the exposed hindwing surface using a pipette. The forewing should be kept in the vertical position during this step.

(5) The vertically held forewing is now placed back onto the solution. The chemical solution is now sandwiched between the fore- and hindwings (see Figure 1a). If the sandwiched chemical solution is not enough, additional 20 μL can be applied from the side at this point, resulting in a total volume of approximately 40 μL. This volume is the maximum to be held between the wings. Then, the whole system is placed in a simple humidified chamber to let chemicals penetrate to wing epithelial cells (see Figure 1b).

(6) After a while, the sandwiched chemical solution is washed out by insect Ringer's solution applied from the side. If the sandwiched chemical solution is brownish at this point, this is caused by bleeding. In this case, an applied chemical is not loaded properly. When this occurs, fluorescent dyes cannot penetrate to the wing tissue and no clear fluorescent observation can be made.

(7) The forewing is picked up again. The flipped forewing does not have to be held vertically at this point. This re-flipping is possible within approximately 2 hours after pupation.

(8) Insect Ringer's solution is applied again to wash out chemicals completely.

(9) Liquid spilled over is wiped out by a piece of paper towel (e.g., Kimwipes™). Approximately 10 μL of insect Ringer's solution is placed onto the hindwing surface. Without this, the forewing would directly stick onto a piece of slide glass in the next step, leading to wrinkle formation and bubble trapping. These in turn may potentially cause artefacts in subsequent observations.

(10) A piece of slide glass is placed onto the hindwing. The plastic film and slide glass are then held together by your fingertips at the circled position.

(11) The whole system, held by your fingertips at the circled position, is now turned upside down. Do not touch the pupa at all.

(12) The plastic film is removed.

(13) Liquid on the slide glass is wiped out by a piece of folded paper towel. If this step is not performed, fluorescent images are not of high quality due to the liquid between the hindwing and slide glass. It is now ready to set the whole system on a stage of inverted microscope.

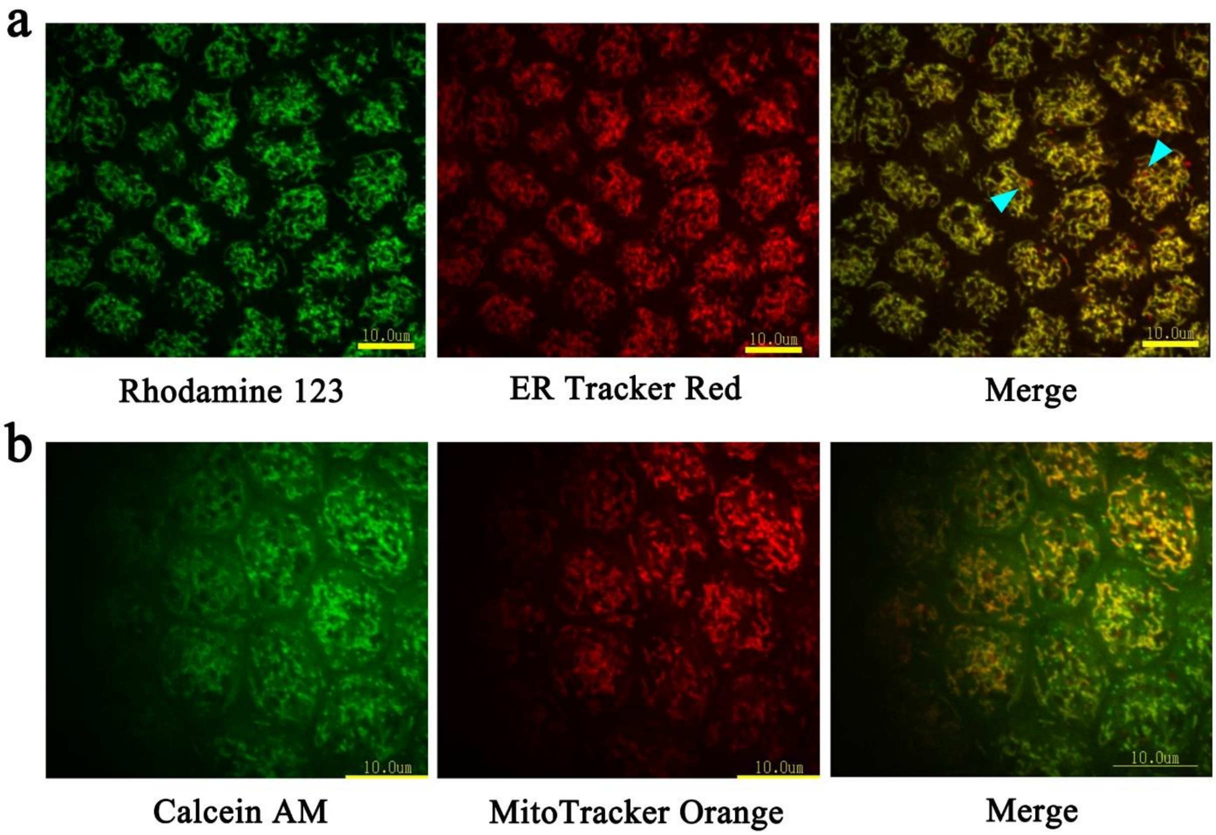

Figure B. Additional double staining patterns of pupal epithelial cells. (a) Double staining with Rhodamine 123 for mitochondria and ER Tacker Red for ER. Specific ER staining dots are indicated by arrowheads. **(b)** Double staining with Calcein AM for cytoplasm and MitoTracker Orange for mitochondria.

Supporting Information References

1. **Kusaba K, Otaki JM** (2009) Positional dependence of scale size and shape in butterfly wings: wing-wide phenotypic coordination of color-pattern elements and background. J Insect Physiol 55: 174-182.
2. **Dhungel B, Otaki JM** (2009) Local pharmacological effects of tungstate on the color-pattern determination of butterfly wings: a possible relationship between the eyespot and parafocal element. Zool Sci 26: 758-764.
3. **Iwata M, Ohno Y, Otaki JM** (2014) Real-time *in vivo* imaging of butterfly wing development: revealing the cellular dynamics of the pupal wing tissue. PLoS ONE 9: e89500.
4. **Ohno Y, Otaki JM** (2015) Spontaneous long-range calcium waves in developing butterfly wings. BMC Dev Biol 15: 17.
5. **Ando T, Fujiwara H** (2013) Electroporation-mediated somatic transgenesis for rapid functional analysis in insects. Development 140: 454-458.
